# Effects of Supplemental Benzoic Acid, Bromelain, Adipic Acid, and Humic Substances on Nitrogen Utilization, Urine pH, Slurry pH, and Manure Odorous Compounds in Pigs

**DOI:** 10.3390/ani14010082

**Published:** 2023-12-25

**Authors:** Seung Bin Yoo, Yoon Soo Song, Siyoung Seo, Beob Gyun Kim

**Affiliations:** 1 Department of Animal Science, Konkuk University, Seoul 05029, Republic of Korea; dbtmdqls0316@konkuk.ac.kr (S.B.Y.); sang8sys@konkuk.ac.kr (Y.S.S.); 2 Animal Environmental Division, National Institute of Animal Science, Wanju 55365, Republic of Korea; seosi@korea.kr

**Keywords:** nitrogen balance, pigs, slurry pH

## Abstract

**Simple Summary:**

Ammonia gases are produced from urea in the urine and slurry due to the action of urease. Odorous compounds are produced by microbial fermentation that utilizes an undigested nutrient in pig slurry. Various additives have been researched to mitigate ammonia emissions or odorous compound production. These studies involve inducing acidic conditions in urine or slurry, enhancing nitrogen utilization, inhibiting urease, or absorbing odorous compounds in pig slurry. However, information about the comparison of these additives is lacking. In this study, we evaluate and compare the effects of each additive on nitrogen utilization, urinary pH, slurry pH, and odorous compound concentrations. The five experimental diets were (1) a control diet, (2) the control diet with 1% benzoic acid, (3) the control diet with 1% adipic acid, (4) the control diet with 1% bromelain, and (5) the control diet with 1% humic substances. Fecal dry matter and nitrogen output were greatest in pigs fed the humic substances diet. Daily retained nitrogen and the nitrogen retention rate tended to be lowest in pigs fed an adipic acid diet. Urinary pH was lowest in pigs fed the adipic acid diet. In addition, slurry pH did not affect odorous compound concentrations, and some odorous compounds tended to be lowest in pigs fed the humic substances diet.

**Abstract:**

The objective was to evaluate the effects of benzoic acid, bromelain, adipic acid, and humic substance supplementation on nitrogen balance, urinary pH, slurry pH, and manure odorous compounds in pigs. Fifteen castrated male pigs with an initial body weight of 37.9 kg (standard deviation = 4.1) were individually housed in metabolism crates. The animals were allocated to a triplicated 5 × 2 incomplete Latin square design with 15 animals, 5 experimental diets, and 2 periods. The basal diet mainly consisted of corn, soybean meal, and rapeseed meal. Four experimental diets were prepared by supplementing each additive at a concentration of 10 g/kg at the expense of corn starch to the basal diet. Each period consisted of a 4-day adaptation period, a 24 h collection period for slurry sampling, and a 4-day collection period for feces and urine. The feces and urine collected for 24 h on day 5 were mixed at a ratio of fecal weight and urine weight to obtain slurry samples. The apparent total tract digestibility N in pigs fed the humic substance diet was the least (*p* < 0.05) compared to the other groups. The daily retained N and N retention as % ingested tended (*p* < 0.10) to be the lowest in the adipic acid group among the treatments. The urinary pH in pigs fed the adipic acid diet was less (*p* < 0.05) than that in other groups except the benzoic acid group. The slurry pH tended to differ among the treatment groups (*p* = 0.074) with the lowest value in the pigs fed the adipic acid diet. The concentrations of indole in slurry (*p* = 0.084) and isovalerate in feces (*p* = 0.062) tended to differ among the groups with the lowest values in the pigs fed the humic substance diet. In conclusion, adipic acid supplementation in pig diets can decrease urinary pH and slurry pH. Although benzoic acid and adipic acid have limited effects in reducing odorous compounds, humic substances have the potential to reduce some odorous compounds.

## 1. Introduction

In the swine industry, the release of ammonia gases and offensive odorous compounds, including volatile fatty acids (VFA) and volatile organic compounds, has been noticed due to environmental issues. Ammonia gases are produced in pig slurry due to the hydrolysis of urea by the action of urease [1]. The emission of ammonia gases results in the formation of fine particulates and the eutrophication of ecosystems [2]. The odorous compounds are generated by the microbial fermentation of undigested nutrients in the slurry [3], and these compounds are potential challenges to public health [4]. Therefore, efforts to reduce odorous compounds emitted from pig slurry are important to ensure sustainable livestock farming. Thus, research on effective strategies for reducing ammonia and odorous compounds has been conducted [5,6,7]. Supplemental organic acids such as benzoic acid (BA), an aromatic carboxylic acid, and adipic acid, a dicarboxylic acid with a six-carbon chain, in swine diets have been reported to be excreted through urine and, consequently, lower the pH of urine to prevent the degradation of urea to ammonia [8,9,10]. In addition, BA has been reported to decrease nitrogen (N) excretion by improving N utilization [11,12]. Supplemental protease is also considered one of the methods to reduce fecal N excretion from pigs by improving protein digestion and absorption [13]. Bromelain is a proteolytic enzyme extracted from pineapple. Humic substances (HS) consisted of humic acid, fulvic acid, and inorganic compounds derived from soil have also been suggested to reduce the ammonia emission from pig manure by inhibiting urease activity [14] and to reduce the odorous compounds in pig manure [15].

Although BA, adipic acid, bromelain, and HS may reduce odorous compounds and ammonia from the slurry in a pig house, the effects of these additives have not been compared. Therefore, the objective of this study was to determine the effects of supplemental BA, adipic acid, bromelain, and HS to diets on the N balance, urinary pH, slurry pH, and odorous compound concentrations in pig manure.

## 2. Materials and Methods

All protocols for the animal experiment were reviewed and approved by the Institutional Animal Care and Use Committee of Konkuk University (Seoul, Republic of Korea, KU23055).

### 2.1. Animals, Experimental Design, and Diets

Fifteen castrated male pigs (Landrace × Yorkshire) with an initial mean body weight of 37.9 kg (standard deviation = 4.1) were allotted to a triplicated 5 × 2 incomplete Latin square design with 15 animals, 5 experimental diets, and 2 periods to obtain 6 observations per treatment. A spreadsheet-based program was used to minimize potential carryover effects [16]. Pigs were individually housed in metabolism crates equipped with a feeder and a nipple drinker. The five dietary treatments were (1) a control diet, (2) the control diet + 1% BA, (3) the control diet + 1% adipic acid, (4) the control diet + 1% bromelain, and (5) the control diet + 1% HS (Table 1). All experimental diets were formulated based on corn, soybean meal, and rapeseed meal. The experimental diets were formulated to meet or exceed the nutrient requirement estimates suggested by the NRC [17]. Each additive was supplemented to the control diet at the expense of corn starch.

### 2.2. Feeding and Sample Collection

Using the body weight of each pig and the metabolizable energy concentrations of the experimental diets, daily feed allotments were calculated at the beginning of each experimental period as 3 times the maintenance energy requirement (i.e., 197 kcal of metabolizable energy per kg of body weight^0.60^) [18]. The amount of feed allotment was divided into 2 equal quantities and provided to pigs at 0800 and 1700 h. Water was freely available at all times.

The experimental period consisted of a 4-day adaptation period, a 24 h collection period for slurry sampling, and a 4-day collection period for fecal and urine collection [19]. The feces and urine collected for 24 h on day 5 were mixed at a ratio of fecal weight and urine weight to obtain slurry samples. On days 6 and 10, chromic oxide was added as a marker at 0.5% to the morning meals. The marker-to-marker procedure was employed for the total collection of feces [19]. The fecal collection started when the green color of chromium began to appear in the feces and ended when the green color appeared again. Urine collection was initiated at 1000 h on day 6 and terminated at 1000 h on day 10. Urine was weighted and collected twice daily at 1000 h and 1900 h. The urinary and slurry pH were measured using a pH meter (PM-2, CAS Inc., Yangju, Gyung-gi, Republic of Korea) immediately after weighing urine. All feces and urine samples were stored at −20 °C immediately after collection.

### 2.3. Volatile Fatty Acid Analyses

For the VFA analysis, 1 mL of a 25% meta-phosphoric acid solution (Sigma-Aldrich, St. Louis, MO, USA) was added to each of 5 g of feces and 5 mL of slurry in a 15 mL plastic tube, and 0.05 mL of a saturated mercury (II) chloride solution (Sigma-Aldrich, St. Louis, MO, USA) was also added to the plastic tube. Then, the solution was centrifuged at 3134× *g* for 20 min at 20 °C, and then 1 mL of supernatant was collected. The supernatant was also centrifuged for 10 min at 13,800× *g* and filtered through a 0.2-μm Whatman filter (Whatman, Uppsala, Sweden). The filtrates were transferred to 2 mL gas chromatography vials (Agilent, Santa Clara, CA, USA). The concentration of VFA was determined using a gas chromatograph (6890N, Agilent, Santa Clara, CA, USA) that was equipped with an HP-INNOWax column (30 m × 0.25 mm × 0.25 μm; Agilent, Santa Clara, CA, USA) and a flame ionization detector. A sample of 0.2 μL was injected at a 10-to-1 split ratio. The gas chromatography oven was initially set at 80 °C for 2 min and increased to 120 °C at a rate of 20 °C per min. The oven temperature was then increased to 205 °C at 10 °C per min, and finally held at 205 °C for 2 min. The injection and detection ports of gas chromatography were maintained at 250 °C.

### 2.4. Phenol and Indole Analyses

After centrifuging the fecal and slurry samples at 3134× *g* for 20 min at 20 °C, 4 mL of supernatant was collected. The supernatant was placed in a 20-mL glass vial, and then 4 mL of chloroform (Merck, Darmstadt, Germany) and 60 μL of 4 M sodium hydroxide solution (Sigma-Aldrich, St. Louis, MO, USA) were also added to the glass vial and mixed with supernatant. The mixture in the glass vial was centrifuged at 3134× *g* for 20 min at 20 °C, and the chloroform layer was collected and placed into a 2.0-mL gas chromatography vial (Agilent, Santa Clara, CA, USA). Phenols and indoles in the chloroform layer were determined using a gas chromatograph (6890N, Agilent, Santa Clara, CA, USA) that was equipped with a DB-1 column (30 m × 0.25 mm × 0.25 μm, Agilent, Santa Clara, CA, USA) and a flame ionization detector. A sample of 2.0 μL was injected at a 5-to-1 split ratio. The gas chromatography oven was initially set at 40 °C for 5 min, increased to 230 °C at a rate of 10 °C per min, and finally held at 230 °C for 2 min. The injection and detection ports of gas chromatography were maintained at 250 °C.

### 2.5. Chemical Analyses

The fecal samples were dried in a forced-air drying oven at 55 °C until constant weight was achieved and ground before analysis. Samples of ingredients, diets, and feces were analyzed for dry matter (DM; [20]) and ash (method 942.05) according to the AOAC [21]. Samples of ingredients, diets, feces, and urine were analyzed for crude protein (method 990.03). Gross energy in the diet samples was determined using bomb calorimetry (Parr 6200, Parr Instruments Co., Moline, IL, USA). Amylase-treated neutral detergent fiber (method 2002.04) and acid detergent fiber (method 973.18) in the diets were analyzed according to the AOAC [21].

### 2.6. Calculations

The apparent total tract digestibility (ATTD) of DM and N was calculated using the following equations:

ATTD of DM (%) = [(DM_intake_ − DM_feces_) ÷ DM_intake_] × 100

ATTD of N (%) = [(N_intake_ − N_feces_) ÷ N_intake_] × 100

where DM_intake_ and DM_feces_ represent the amount of DM intake (g/d) and fecal DM output (g/d), respectively, and N_intake_ and N_feces_ represent the amount of N intake (g/d) and fecal N output (g/d), respectively. The balance of N in pigs fed experimental diets was calculated using the following equations:

N retention rate as % of ingested (%) = 100 × (N_intake_ − N_feces_ − N_urine_) ÷ N_intake_

N retention rate as % of digested (%) = 100 × (N_intake_ − N_feces_ − N_urine_) ÷ (N_intake_ − N_feces_)

where N_intake_, N_feces,_ and N_urine_ represent the amount of N intake (g/d), fecal N output (g/d), and urinary N output (g/d), respectively.

### 2.7. Statistical Analyses

Experimental data were statistically analyzed using the MIXED procedures of SAS (SAS Inst. Inc., Cary, NC, USA). The statistical model included the experimental diet as the fixed variable and replication and period within replication as the random variables. Least-square means were calculated, and the means were separated using the PDIFF option. An individual pig was the experimental unit, and statistical significance and tendency were declared at *p* < 0.05 and 0.05 ≤ *p* < 0.10, respectively.

## 3. Results

All the pigs were in good health throughout the experiment and readily consumed their daily feed allowance. The ATTD of DM and N in pigs fed the HS diet was less (*p* < 0.05) than those fed the control diet (Table 2). The daily retained N and N retention as % ingested tended (*p* < 0.10) to be the greatest in the BA group and the lowest in the adipic acid group among the treatments.

The urinary pH in pigs fed the adipic acid diet was less (*p* < 0.05) than the control, the bromelain group, and the HS group but did not differ from the BA diet (Table 3). The slurry pH in pigs fed the adipic acid diet tended to be the lowest (*p* = 0.074) among experimental diets.

The VFA concentrations in feces and slurry were not different among pigs fed the experimental diets (Table 4). However, the isovalerate concentrations in feces (*p* = 0.062) and the indole in slurry tended to be the lowest (*p* = 0.084) in pigs fed the HS diet among experimental diets.

## 4. Discussion

Benzoic acid is one of the organic acids that have been reported to improve the environment of the gastrointestinal tract and growth performance [22,23]. Patráš et al. [24] reported a tendency for N retention to improve due to 1% dietary BA inclusion. Murphy et al. [25] also reported a linear increase in N retention in response to feeding BA at 0, 1%, 2%, and 3% to pigs. The supplementation of organic acids has been suggested to improve N utilization by lowering the pH in the stomach [26,27]. Pepsin is activated at an acidic condition, and thus, supplemental acids can improve N digestibility by lowering gastric pH, which likely affects N metabolism in pigs [28]. In this study, however, supplemental BA did not affect N balance, which is inconsistent with the previous studies. The reason for the inconsistency among the studies remains unclear, but the different ingredient compositions in diets potentially resulted in varying acid-binding capacities of diets in the stomach [29]. Patráš et al. [24] used 43 to 57% corn, 30% wheat, and 8 to 22% soybean meal as the main ingredients and Murphy et al. [25] used 35 to 38% wheat, 25% barley, 17% soybean meal, and 15% corn, whereas the diets in the present experiment were based on 68% corn, 17% soybean meal, and 10% rapeseed meal. Corn has been suggested to have a greater acid-binding capacity compared with wheat [29], which may have resulted in the lack of effects of BA on N balance in the present work. Furthermore, this suggestion is supported by Bühler et al. [30] who reported that dietary BA did not have an effect on the N balance in pigs fed a diet containing 20% barley, which has a greater acid-binding capacity compared with corn or wheat. Additionally, variations in experimental conditions can also potentially affect the effects of supplemental BA in pigs.

The decreases in daily retained N and the N retention rate of the adipic acid group in the present study may be explained by the toxic properties of adipic acid [31]. Administering adipic acid to rats induced an increased lavage protein in their bodies [31], which suggests that adipic acid in animal bodies may act as a toxic material. Adverse effects of supplemental adipic acid on the growth performance of pigs [28] and lysine utilization in pigs [32] have also been reported. Dietary adipic acid appears to decrease N utilization in pigs due to additional protein synthesis for eliminating adipic acid toxicity.

Bromelain is a protease that breaks down polypeptide compounds. Bromelain has been reported to improve N digestibility, and consequently, reduce fecal N excretion and noxious gas emission in nursery pigs [33,34]. In the present work, however, supplemental bromelain did not improve N digestibility in growing pigs, which is likely due to the different growth stages of pigs. Mc Alpine et al. [35] also failed to find the effects of supplemental protease on N digestibility in finishing pigs. It appears that a sufficient quantity of protease is secreted for the digestion of dietary protein in growing and finishing pigs but not in nursery pigs based on the greater protein digestibility in older pigs compared with nursery pigs [36,37]. Therefore, the lack of positive effects of bromelain on N digestibility in the present work is reasonable. In addition, Nguyen et al. [34] also reported that supplemental bromelain or other protease showed positive effects on N digestibility of nursery pigs but not in growing and finishing pigs.

Humic substances consist of humic acids, fulvic acids, and several inorganic substances derived from the soil or sediment. Humic substances themselves contain significant amounts of N, which is not easily degraded [38]. This may partially explain the low N digestibility in the HS group in the present study. Additionally, HS can bind various materials such as minerals and amino acids in diets [39,40], resulting in the inhibition of enzymatic digestion or microbial action against these nutrients. In agreement with the present results, Písaříková et al. [40] noted a 6% reduction in N digestibility via the supplementation of 3% sodium humate in pigs.

Most previous studies reported a reduction in urinary pH by supplemental BA [24,25,30,41,42]. Supplemental BA in pig diets is known to decrease urinary pH as BA is converted into hippuric acid in the liver and excreted in the urine [43]. However, the effect of supplemental BA on urinary pH was not significant in the present work. The inconsistency between this study and the literature may be partially attributed to the different methods used for urine collection. The urine samples were collected directly from the bladder after euthanizing pigs [41,44] or through an equipped catheter [24,45] in previous studies. In the present work, in contrast, urine was collected in containers, and time-based sampling was conducted 2 times per day during collection periods, allowing for more exposure of urine stored in containers to external factors such as air and microbial action [30]. Additionally, water intake may also have contributed to the hippuric acid concentration and pH of urine. Indeed, a positive correlation between the weight of urine and urinary pH was observed in pigs fed the BA diet in the present work (r = 0.39 and *p* = 0.002). Further research is warranted to investigate the interaction between water intake and urinary pH.

Adipic acid is a type of dicarboxylic acid consisted of six carbon atoms derived from longer-chained dicarboxylic acids after beta-oxidation reactions [46]. Adipic acid ingested by pigs is excreted per se or after degradation into shorter dicarboxylic acids through the urine [46], resulting in decreased urinary pH and slurry pH [47]. In agreement, the urinary pH and slurry pH were reduced by supplemental adipic acid in the present study.

Odorous compounds in pig manure are generally produced by microbiota that decompose manure [48]. As microbial genera in manure thrive under an environment of optimum pH, modifying manure pH can weaken microbial action and decrease odorous compound production. Jensen et al. [49] observed decreases in indole and skatole production when modifying the pH of pig slurry below 5 or above 8 by deactivating microbes. Zhu and Jacobson [48] also suggested that the optimal pH for microbial growth ranges from 5.0 to 8.5. The slurry pH in all treatment groups in the present study was within the range that provides the optimal environment for microbiota, supporting the lack of difference in odorous compound concentrations among treatment groups, except indole in the HS group.

The observation of no decrease in odorous compound concentrations in feces by supplemental bromelain is most likely due to the lack of changes in N digestibility. Most odorous compounds are produced from undigested amino acids or N compounds [6,50,51].

The tendency for the reduction in isovalerate in feces and indole in slurry from pigs fed the HS diet remains unclear because other odorous compounds were not affected by supplementing HS. In a previous study, however, *Propionibacterium* genera were reported to utilize humic acid as a terminal electron acceptor in the bacterial culture medium [52]. Benz et al. [53] also observed that *Propionibacterium freudenreichii* degraded propionate to acetate by using humic acid as a terminal electron acceptor. Therefore, the presence of HS might have encouraged *Propionibacterium* to further degrade isovalerate to produce lower-molecule compounds, resulting in the decreased isovalerate concentration in the present study. The role of HS might also partially explain the decrease in the indole concentration of slurry because indole can be degraded by anaerobic microbial fermentation [54]. Further research is warranted to investigate the mechanisms of HS during anaerobic microbial fermentation against odorous compounds.

## 5. Conclusions

The effects of supplemental additives on nitrogen utilization, urinary pH, slurry pH, and odorous compounds varied. Supplementing benzoic acid at 1% did not decrease urinary pH and slurry pH, whereas adipic acid at 1% lowered urinary pH in growing pigs. Supplementing humic substances at 1% negatively affected the nutrient digestibility but tended to decrease some odorous compound concentrations in feces and slurry. Overall, supplemental adipic acid was beneficial for lowering urinary pH and humic substances were effective in reducing odorous compounds in pig manure.

## Figures and Tables

**Table 1:** Ingredient composition and chemical composition of experimental diets on an as-is basis.

Item	Experimental Diet				
	Control	Benzoic Acid	Adipic Acid	Bromelain	Humic Substances ^1^
Ingredients, %					
Ground corn	68.3	68.3	68.3	68.3	68.3
Soybean meal, 45.1% crude protein	17.0	17.0	17.0	17.0	17.0
Rapeseed meal	10.0	10.0	10.0	10.0	10.0
Soybean oil	1.0	1.0	1.0	1.0	1.0
L-Lys·HCl, 78.8%	0.32	0.32	0.32	0.32	0.32
L-Thr, 99.0%	0.07	0.07	0.07	0.07	0.07
DL-Met, 99.0%	0.02	0.02	0.02	0.02	0.02
Dicalcium phosphate	1.0	1.0	1.0	1.0	1.0
Ground limestone	0.8	0.8	0.8	0.8	0.8
Salt	0.3	0.3	0.3	0.3	0.3
Vitamin-mineral premix ^2^	0.3	0.3	0.3	0.3	0.3
Corn starch	1.0	-	-	-	-
Benzoic acid	-	1.0	-	-	-
Adipic acid	-	-	1.0	-	-
Bromelain	-	-	-	1.0	-
Humic substances	-	-	-	-	1.0
Analyzed chemical composition					
Dry matter, %	87.5	87.0	87.5	87.5	87.4
Gross energy, kcal/kg	3887	3907	3899	3900	3868
Crude protein, %	17.4	17.5	17.4	17.5	18.0
Ash, %	5.1	5.0	5.0	5.2	5.5
Amylase-treated neutral detergent fiber, %	10.4	10.5	11.1	9.5	10.9
Acid detergent fiber, %	4.1	4.2	4.4	3.8	4.2

^1^ Humic substances contained 55.8% humic acid, 2.7% fulvic acid, and 9.5% potassium oxide, as-is basis. ^2^ Provided the following quantities per kilogram of complete diet: vitamin A, 18,000 IU; vitamin D_3_, 3600 IU; vitamin E, 60 IU; vitamin K, 5 mg; thiamin, 5 mg; riboflavin, 8 mg; pyridoxine, 5 mg; vitamin B_12_, 0.06 mg; pantothenic acid, 30 mg; folic acid, 2 mg; niacin, 30 mg; biotin, 0.30 mg; Co, 0.75 mg as cobalt sulfate; Cu, 60 mg as copper sulfate; Fe, 120 mg as iron sulfate; I, 0.68 mg as calcium iodate; Mn, 60 mg as manganese sulfate; Zn, 60 mg as zinc sulfate.

**Table 2:** Apparent total tract digestibility (ATTD) of dry matter (DM) and nitrogen (N) balance in pigs fed the experimental diets ^1^.

Item	Experimental Diet ^2^					SEM	*p*-Value
	Control	Benzoic Acid	Adipic Acid	Bromelain	Humic Substances		
Dry matter intake, kg/d	1.4	1.4	1.4	1.4	1.4	0.06	0.888
N intake, g/d	46.5	46.9	46.9	46.5	49.0	2.02	0.124
Fecal DM output, kg/d	0.18 ^bc^	0.19 ^ab^	0.19 ^bc^	0.17 ^c^	0.21 ^a^	0.01	0.006
ATTD of DM, %	88.2 ^ab^	87.1 ^bc^	87.5 ^abc^	88.5 ^a^	86.3 ^c^	1.01	0.022
Fecal N output, g/d	6.0 ^b^	6.4 ^b^	6.5 ^b^	5.7 ^b^	7.9 ^a^	0.51	0.002
ATTD of N, %	86.9 ^a^	86.2 ^a^	86.0 ^a^	87.6 ^a^	83.7 ^b^	1.01	0.018
Urine output, kg/d	2.4	2.7	3.0	3.3	3.1	0.36	0.334
Urinary N output, g/d	14.2	13.3	17.8	15.7	16.2	2.04	0.158
Digested N, g/d	40.5	40.5	40.4	40.8	41.0	2.03	0.980
Retained N, g/d	26.2	27.2	22.7	25.0	24.8	1.57	0.098
N retention, % of ingested	56.7	58.2	48.3	54.0	50.8	3.59	0.074
N retention, % of digested	65.4	67.6	56.4	61.7	60.7	4.02	0.144

^1^ Data are least squares means of 6 observations. ^2^ Each of benzoic acid, adipic acid, bromelain, and humic substances was supplemented to the control diet at 1% at the expense of corn starch. ^a–c^ Means within a row without a common superscript letter differ (*p* < 0.05).

**Table 3:** Urinary and slurry pH of pigs fed the experimental diets ^1^.

Item	Experimental Diet ^2^					SEM	*p*-Value
	Control	Benzoic Acid	Adipic Acid	Bromelain	Humic Substances		
Urinary pH	8.61 ^a^	7.83 ^ab^	7.17 ^b^	8.59 ^a^	8.67 ^a^	0.30	0.006
Slurry pH	8.06	7.55	7.25	7.93	8.04	0.31	0.074

^1^ Data are least squares means of 6 observations. ^2^ Each of benzoic acid, adipic acid, bromelain, and humic substances was supplemented to the control diet at 1% at the expense of corn starch. ^a,b^ Means within a row without a common superscript letter differ (*p* < 0.05).

**Table 4:** Volatile fatty acids (VFA), phenol compounds, and indole compounds in feces and slurry from pigs fed the experimental diets ^1^.

Item, mg/L	Experimental Diet ^2^					SEM	*p*-Value
	Control	Benzoic Acid	Adipic Acid	Bromelain	Humic Substances		
VFA in feces							
Acetate	3930	3868	3559	3943	3641	283	0.698
Propionate	1981	1807	1788	1742	1741	182	0.818
Isobutyrate	290	264	247	218	201	29	0.153
Butyrate	1097	842	984	949	1030	116	0.607
Isovalerate	490	417	407	366	322	43	0.062
Valerate	529	402	406	346	361	66	0.139
Phenol compound in feces ^3^							
*p*-Cresol	16.0	14.5	20.0	18.1	11.7	3.6	0.431
Indole compound in feces							
Indole	5.5	7.5	5.1	3.2	4.0	1.4	0.485
Skatole	9.0	7.3	10.5	8.8	6.8	2.2	0.160
VFA in slurry							
Acetate	1268	1340	1220	1238	1003	177	0.727
Propionate	387	418	482	344	314	86	0.650
Isobutyrate	56.7	57.2	64.9	41.8	35.2	13.1	0.445
Butyrate	243	220	261	182	191	53	0.787
Isovalerate	102	95	115	76	61	22	0.447
Valerate	118	101	114	68	63	29	0.437
Phenol compound in slurry							
Phenol	4.5	4.6	3.1	4.7	3.9	0.8	0.512
*p*-Cresol	155	136	84	109	78	38	0.209
Indole compound in slurry							
Indole	1.6	1.7	1.2	0.8	0.7	0.4	0.084
Skatole	1.5	1.0	1.3	1.0	0.8	0.3	0.290

^1^ Data are least squares means of 6 observations. ^2^ Each of benzoic acid, adipic acid, bromelain, and humic substances was supplemented to the control diet at 1% at the expense of corn starch. ^3^ Phenol was not detected in the fecal samples.

## Data Availability

The data presented in the current work are available.
